# The Patient-Reported Outcomes Measurement Information System perspective of adults with long-standing atypical hemolytic uremic syndrome

**DOI:** 10.1016/j.rpth.2025.103224

**Published:** 2025-10-13

**Authors:** Anne Hubben, Jenna Brown, Linda Burke, Len Woodward, Clare Martin, Keith McCrae, Shruti Chaturvedi

**Affiliations:** 1 Department of Hematology and Medical Oncology, Cleveland Clinic Foundation, Cleveland, Ohio, USA; 2 Department of Hematology and Medical Oncology, Johns Hopkins University, Baltimore, Maryland, USA; 3 aHUS Alliance Global Action, Knutsford, England, United Kingdom; 4 The United States Thrombotic Microangiopathy Alliance, Columbus, Ohio, USA

**Keywords:** atypical hemolytic uremic syndrome, health-related quality of life, microangiopathic hemolytic anemia, patient reported outcomes, thrombosis, thrombotic microangiopathies

## Abstract

**Background:**

Atypical hemolytic uremic syndrome (aHUS) is a thrombotic microangiopathy driven by a dysregulation of the complement system, disproportionately affecting the kidneys. While terminal complement blockade has reduced the risk of death and progression to end stage renal disease, patients face significant chronic health challenges. The patient experience in the current treatment era has not been well-characterized.

**Objectives:**

To evaluate patient-reported health outcomes (PRO) across global health domains, including physical and cognitive function and social isolation.

**Methods:**

We performed a cross-sectional analysis of adults with aHUS using validated National Institute of Health Patient-Reported Outcomes Measurement Information System instruments.

**Results:**

Compared with a normative reference population, our aHUS cohort (*N* = 50) had significantly lower mean ± SD T-scores for physical function (44.6 ± 8.9; *P* < .001) and cognitive function (41.4 ± 12.0; *P* < .001), and higher scores for anxiety (59.2 ± 9.7; *P* < .001), depression (55.8 ± 9.7; *P* < .001), fatigue (59.3 ± 10.7; *P* < .001), and sleep disturbance (53.6 ± 7.5, *P* < .001), suggesting overall worse functioning in several domains. Patients with self-reported anxiety or depression had higher scores for depression and anxiety and worse functioning across all measured outcomes.

**Conclusion:**

In this first study of PROs in patients with long-standing aHUS in the era of complement-directed therapies, patients reported a chronically impaired global health status with reduced physical and cognitive function and higher levels of fatigue, anxiety, depression, and sleep disturbances compared with the general population. Our data underscore that aHUS is a multisystem disease that may be associated with persistent symptoms even during periods of hematologic and renal quiescence.

## Essentials

• Adults with aHUS experience chronic health issues despite advanced therapies.
• In this study, quality of life outcomes were characterized using the National Institutes of Health Patient-Reported Outcomes Measurement Information System (PROMIS) metrics.
• Patients had impaired physical and cognitive function, fatigue, anxiety, and depression.
• These findings highlight the unmet needs in addressing quality of life for aHUS patients.

## 1 Introduction

Atypical hemolytic uremic syndrome (aHUS) is a thrombotic microangiopathy (TMA) driven by dysregulation of the complement system and characterized by microangiopathic hemolytic anemia, thrombocytopenia, and microvascular ischemic injury that disproportionately affects the kidneys [1]. Historically, in the era prior to anticomplement therapies, aHUS was associated with mortality approaching 25% [2] and a higher morbidity with progression to end-stage renal disease (ESRD) in more than 50% of affected patients [3,4]. Terminal complement system blockade has dramatically improved patient outcomes and changed the natural history of aHUS. Eculizumab, approved in 2011, and the longer-acting ravulizumab, approved in 2019, are highly effective anti-C5 monoclonal antibodies that induce rapid and sustained renal and hematologic recovery in aHUS, as well as improved Functional Assessment of Chronic Illness Therapy (FACIT)-fatigue scores in early clinical trials [[5], [6], [7]]. The risk of death and ESRD has reduced from 50% to 6% to 15% at 1 to 2 years [5]. Despite these improvements, patients with aHUS face ongoing health challenges. A recent cohort study from the United Kingdom reported that, despite treatment with C5 inhibition, many patients had persistent severe chronic kidney disease (CKD), with 46% of patients having CKD stage ≥ 3 and 21% of patients having ESRD [8]. Follow-up studies of patients in the registrational trials of complement inhibitors also reported that nearly a third of patients experience a decline in renal function over time [[9], [10], [11]]. Many patients need to continue therapy long-term, and others experience the risk of aHUS relapse. Finally, patients with aHUS may experience the challenges of living with a rare disease, which can be isolating and impact well-being and quality of life [12,13].

Patient-reported outcomes (PRO) are a valuable method for capturing relevant outcomes in patient-centered clinical research and highlighting areas of unmet need in the management of rare diseases [14]. While treatment with terminal complement inhibition has improved hematologic and renal outcomes in aHUS, the patient experience and symptom burden in the current treatment era have not been characterized. We conducted a cross-sectional study of adult patients with long-standing aHUS to evaluate patient-reported health outcomes in the current treatment era.

## 2 Methods

### 2.1 Participants and recruitment

Adults with a self-reported established diagnosis of aHUS were recruited for this cross-sectional study. Participants were recruited by posting the institutional review board-approved survey link to the social media pages of the aHUS Alliance Global Action, an international nonprofit organization dedicated to supporting patients and families affected by aHUS, with 3200 followers, and the United States Thrombotic Microangiopathy Alliance, a United States-based patient support and advocacy organization dedicated to TMA, with approximately 1000 followers. The survey was also disseminated via the email mailing list of the United States Thrombotic Microangiopathy Alliance. The survey was administered for 2 months from November 27, 2023, to January 26, 2024. While the study involved minimal risk, participants were instructed to stop taking the survey at any point if they experienced emotional distress and were also directed to a 24-hour mental health helpline. Online consent was obtained prior to administering the survey. Only patients aged ≥18 years were included, and caregivers or proxy responders for adolescents were not included. Survey responses were collected and managed in Research Electronic Data Capture (REDCap), a secure web-based application for building and managing online research surveys and databases hosted by the United States Thrombotic Microangiopathy Alliance consortium. The Institutional Review Board at Johns Hopkins University approved this study.

### 2.2 Survey instruments

The survey tool included the National Institutes of Health (NIH) Patient-Reported Outcomes Measurement Information System (PROMIS) instruments (PROMIS-29v2.0) to assess 7 global health domains (including physical function, fatigue, pain, depression, anxiety, participation in social roles, and sleep disturbance) [15], as well as cognitive function (Cognitive Function 8a) and social isolation (Social Isolation 4a). We selected the PROMIS instruments because normative data from large populations are available, and these are validated NIH common data elements that can be compared across existing and future studies. PROMIS measures are scored on a T-score metric, where the mean of the normative reference population is 50 and the SD is 10. Higher scores indicate that more of the concept is being measured. Therefore, lower scores are worse for measures such as physical function, cognitive function, and social participation, while higher scores are worse for measures such as anxiety, depression, fatigue, pain, sleep disturbance, and social isolation. The specific PROMIS instruments included were chosen based on input from clinicians and representatives from the aHUS Global Alliance.

In addition to the PROMIS instruments, the survey included questions on self-reported medical history, including diagnosis, treatments, and outcomes of aHUS (relapse and renal failure), medication history, and daily symptoms. Prior to dissemination, the survey was pilot-tested and edited based on input from 5 volunteer participants with aHUS who administered the survey and interviewed for feedback on the themes included, ease of comprehension, and ease of self-administration. Patients were also invited to provide narrative written feedback after completing the survey.

This study was conducted as an elective, self-administered, deidentified survey, and medical histories could not be independently verified through medical records. These data were limited by heterogeneity in the study cohort and subject to volunteer and reporting biases. Additionally, important clinical variables could not be controlled for, including the intensity of clinical follow-up, the presence of ongoing triggers for complement activation, verification of clinical vs laboratory remission, adherence to guidelines for complement inhibitor use or discontinuation, and the potential confounding effect of medical comorbidities on patient-reported outcomes. Despite these limitations, the study provides important insights into the subjective patient experience, capturing symptom burden, functional impact, and quality of life in adults living with long-standing aHUS. The use of validated NIH PROMIS instruments enhances the reliability of these findings.

### 2.3 Statistical analysis

Continuous variables were reported as median with IQR or mean ± SD, and categorical variables as counts and percentages. We used a single-sided *t*-test to compare T-scores of these instruments with the population norm and the Mann–Whitney U-test to compare groups based on relapse status and comorbidities, including self-reported CKD and anxiety or depression. *P* < .05 was considered significant.

## 3 Results

### 3.1 Participant characteristics, aHUS history, and current symptom burden

A total of 61 individuals responded to the survey, but only 50 individuals completed at least 1 PRO instrument in its entirety, and this group was analyzed as the study population. The demographic and aHUS-related clinical details of the participant cohort are summarized in Table 1. The median (IQR) age was 43 (34, 54) years; 43 (86%) participants self-identified as White, and 43 (86%) were female. The median (IQR) time from self-reported aHUS diagnosis was 50 (21, 64) months, and 44 (88%) participants reported confirmatory genetic testing to support the aHUS diagnosis. At diagnosis, 98% (49 of 50) of participants had been treated with complement inhibition. Two patients initially received ravulizumab, while the other 47 were started on eculizumab; 28% subsequently switched from eculizumab to ravulizumab. At the time of the survey, 82% (41 of 50) of participants were on complement inhibition (19 eculizumab and 22 ravulizumab). The remainder were no longer on complement inhibition; however, the details of complement therapy discontinuation, remission status, and whether guidelines for discontinuation were followed are unknown. The majority (72%) of participants required dialysis following diagnosis, 10% remained on dialysis when surveyed, and 24% had undergone a kidney transplant. Sixty percent reported CKD; however, the distribution of CKD stages could not be determined due to limitations of self-reported data. Ten participants (20%) reported 1 or more aHUS relapses, 70% reported no relapses, and 10% did not know if they had relapsed and were not included in this subanalysis. At the time of the study, 88% of respondents reported that their disease was under “good” subjective control or in remission. The study did not clarify whether “good control” referred to clinical symptoms or knowledge of objective laboratory parameters such as complete blood counts, lactate dehydrogenase, or percent change in creatinine. Remission status was not independently verified due to the self-reported and survey-based study design.

**Table 1:** Participant demographics and clinical characteristics of atypical hemolytic uremic syndrome.

Characteristic/variable	Study population (*N* = 50) *n* (%)
*Demographics*	
Current age (y), median (IQR)	**43 (34, 54)**
Female sex	43 (86)
Self-identified race/ethnicity	
Black	1 (2)
White	43 (86)
Hispanic	3 (6)
Native American	1 (2)
Other	1 (2)
Prefer not to answer	2 (4)
Time since aHUS diagnosis (y), median (IQR)	50 (21, 64)
*aHUS history*	
Genetic testing for aHUS completed	44 (88)
Treatments received (ever)	
Plasmapheresis	35 (70)
Eculizumab	47 (94)
Ravulizumab	27 (54)
Dialysis	36 (72)
Blood transfusion	2 (4)
Rituximab	3 (6)
I do not know	1 (2)
What treatments are you currently receiving for aHUS	
Plasmapheresis	1 (2)
Eculizumab	19 (38)
Ravulizumab	22 (44)
Dialysis	5 (10)
Other	5 (10)
If you were previously taking eculizumab or ravulizumab, have you?	
Switched from eculizumab to ravulizumab	14 (28)
Switched from ravulizumab to eculizumab	0 (0)
Stopped treatment	8 (16)
Have you received a kidney transplant	12 (24)
Is your aHUS under good control or in remission? (%)	
Yes	44 (88)
No	1 (2)
I do not know	5 (10)
Have you ever experienced aHUS elapse	
Yes	10 (20)
No	35 (70)
I do not know	5 (10)

aHUS, atypical hemolytic uremic syndrome.

### 3.2 The burden of comorbidities and symptoms is high in long-standing aHUS

Patients reported the following symptom burden when asked about their symptoms over the preceding 4 weeks: fatigue (82%), headaches (56%), drowsiness (50%), confusion or foggy thinking (48%), difficulty remembering things (46%), nausea (48%), stomach pain (32%), diarrhea (32%), and swelling of the lower extremities (22%). Joint pain, unexplained bruising, and bone pain were also reported (2% each; Table 2). A majority of respondents reported hypertension (74%) and CKD (60%), while 56% also reported experiencing depression or anxiety – comorbidities that may contribute to chronic symptom burden. All patients were being followed by a healthcare provider for their aHUS with a frequency ranging from more than once a month to once a year, with the largest group reporting quarterly follow-ups. Of the 11 patients who reported being monitored at least once a month, 1 was no longer on any treatment but had noted 10 months of previous eculizumab treatment. It is possible this patient was undergoing more frequent monitoring due to discontinuation of anti-C5 therapy, as most experts advocate for more frequent monitoring after discontinuation [16]. Of the remaining 10 patients with frequent monitoring, 4 had a history of renal transplant and remained on complement inhibition (2 on ravulizumab and 2 on eculizumab), 2 were on dialysis and remained on complement inhibition, and 4 were on ravulizumab alone. None of the patients undergoing monthly or more frequent monitoring had a history of relapse, and the reasons for a more frequent monitoring schedule were not evident. Of those who provided details on their experiences with healthcare, 33 (66%) were followed by a hematologist, 16 (32%) by a nephrologist, and 1 (2%) by a primary care provider. Only a minority (34%; 14 of 41) stated that their chronic symptoms and concerns were acknowledged and recognized by their medical team at above average to complete understanding.

**Table 2:** Symptom burden, comorbidities, and experiences with healthcare.

Question	*N* = 50 *n* (%)
*Symptom burden: in the past 4 wk, were you bothered by?*	**-**
Fatigue or feeling drained	41 (82)
Nausea or upset stomach	24 (48)
Drowsiness or difficulty staying awake	25 (50)
Swelling of legs, ankles, or feet	11 (22)
Stomach pain	16 (32)
Confusion or foggy thinking	24 (48)
Diarrhea	16 (32)
Headaches	28 (56)
Difficulty remembering things	23 (46)
*Comorbidities: which of the following medical issues do you have?*	
Hypertension	37 (74)
Seizures or other neurological conditions	1 (2)
Kidney disease or dialysis	30 (60)
Hyperlipidemia	11 (22)
Lung disease	1 (2)
Heart disease	2 (4)
Stroke	3 (6)
Depression or anxiety	28 (56)
*Are you primarily treated by a PCP or specialist for aHUS?*	
Primary care	1 (2)
Hematologist	33 (66)
Nephrologist	16 (32)
*Do you feel that your medical team is adequately informed about your disease?*	
0 – uninformed	3 (6)
1	6 (12)
2	8 (16)
3	12 (24)
4	11 (22)
5–expert	7 (14)
No answer	3 (6)
*Do you feel that your chronic symptoms are recognized/acknowledged/understood by your medical team?*	
0–not understood	7 (14)
1	3 (6)
2	7 (14)
3	10 (20)
4	11 (22)
5 – completely understood	3 (6)
No answer	9 (18)

aHUS, atypical hemolytic uremic syndrome; PCP, primary care physician.

### 3.3 Participants with aHUS report reduced quality of life in multiple domains

Among the 50 participants included in the study, all 50 completed the PROMIS-29v2.0 instrument, 43 completed the Cognitive Function 8a instrument, and 42 completed the Social Isolation 4a instrument. Compared with a normative reference population (mean ± SD, 50 ± 10), our aHUS cohort had significantly lower mean ± SD T-scores for physical function (44.6 ± 8.9; *P* < .001) and cognitive function (41.4 ± 12.0; *P* < .001), and higher scores for anxiety (59.2 ± 9.7; *P* < .001), depression (55.8 ± 9.7; *P* < .001), fatigue (59.3 ± 10.7; *P* < .001), and sleep disturbance (53.6 ± 7.5; *P* < .001), suggesting overall worse functioning in several domains (Table 3 and Figure). Mean T-scores for pain interference (47.9 ± 9.5; *P* = .249), ability to participate in social roles (51.8 ± 10.2; *P* = .14), and social isolation (49.9 ± 10.7; *P* = .945) were not significantly different from those of the controls. Among the patients with knowledge of their relapse history, those who had experienced 1 or more relapses had lower mean T-scores for physical function (39.2 ± 5.4 vs 46.4 ± 8.8; *P* = .03) and social participation (42.7 ± 4.9 vs 49.3 ± 9.3; *P* = .048) compared with patients without a history of relapse. Scores of all other measures were not significantly different between patients with and without a history of relapse (Supplementary Table S1).

**Table 3:** Scores of atypical hemolytic uremic syndrome patients on selected Patient-Reported Outcomes Measurement Information System instruments and comparison with the normative mean.

PROMIS measure	*N* = 50 T-score, mean (SD)	*P*^a^
*PROMIS-29v2.0*		
Physical function	44.6 (8.9)	<.001
Anxiety	59.2 (9.7)	<.001
Depression	55.8 (9.7)	<.001
Fatigue	59.3 (10.7)	<.001
Sleep disturbance	53.6 (7.5)	.001
Pain interference	47.9 (9.5)	.249
Ability to participate in social roles	51.8 (10.2)	.14
Cognitive function abilities	46.9 (7.1)	.003
Cognitive function 8a	41.4 (12.0)	<.001
Social isolation	49.9 (10.7)	.945

PROMIS, Patient-Reported Outcomes Measurement Information System.

a *P* values are from one-sided *t*-tests compared with normative data with a mean (SD) of 50 (10).

**Figure:**
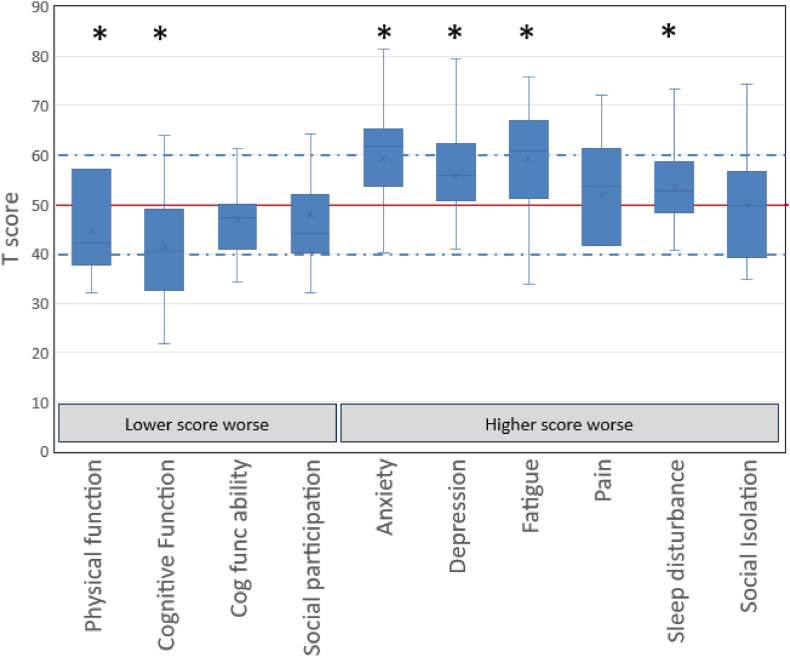
Individuals with atypical hemolytic uremic syndrome have worse scores on patient reported outcomes instruments evaluating multiple domains of quality of life. Compared with a normative reference population (T-score mean ± SD, 50 ± 10), our atypical hemolytic uremic syndrome cohort had significantly higher scores for anxiety, depression, fatigue, and sleep disturbance and lower scores for physical and cognitive function, suggesting overall worse patient-reported functioning in several domains.

### 3.4 Comorbidities associated with worse PROs in aHUS

T-scores of the PRO instruments were not significantly different between participants with self-reported CKD and those with no CKD, or between patients who received dialysis at any point after diagnosis and those who never received dialysis (Supplementary Tables S2 and S3, respectively), although the sample sizes were small. Participants currently receiving hemodialysis had significantly worse scores for physical function; while scores for other domains, such as fatigue, anxiety, depression, and ability to participate in social roles, were numerically worse in this group, these differences were not statistically significant (Supplementary Table S4). However, patients who self-reported a diagnosis of anxiety or depression not only had higher scores for depression and anxiety but also had worse scores for all other PRO measures administered, including physical function, fatigue, sleep disturbance, ability to participate in social roles, pain, cognitive function, and social isolation (Table 4).

**Table 4:** Scores of atypical hemolytic uremic syndrome patients with and without a self-reported diagnosis of anxiety and/or depression on selected Patient-Reported Outcomes Measurement Information System instruments.

PROMIS measure	Self-reported diagnosis of depression and/or anxiety T-score, mean (SD)		*P*^a^
	No	Yes	
*PROMIS-29v2.0*			
Physical function	49.7 (7.4)	40.6 (7.8)	<.001
Anxiety	54.2 (11.7)	63.1 (5.3)	<.001
Depression	51.0 (10.2)	59.6 (7.4)	.001
Fatigue	52.6 (9.8)	64.3 (8.2)	<.001
Sleep disturbance	50.5 (5.7)	56.1 (7.9)	.008
Pain interference	45.3 (6.6)	56.5 (9.8)	<.001
Ability to participate in social roles	54.5 (7.8)	43.5 (7.7)	<.001
Cognitive function abilities	49.9 (7.6)	44.8 (6.1)	.018
Cognitive function 8a	49.9 (10.6)	35.9 (9.5)	<.001
Social isolation	42.5 (8.9)	54.9 (8.9)	<.001

PROMIS, Patient-Reported Outcomes Measurement Information System.

a *P* values were calculated using the Mann–Whitney U-test to compare groups.

## 4 Discussion

In this first study of PROs in patients with long-standing aHUS in the era of complement-directed therapies, patients reported a chronically impaired global health status with reduced physical and cognitive function and higher levels of fatigue, anxiety, depression, and sleep disturbances compared with the general population.

Terminal complement inhibition has transformed acute outcomes of aHUS, reducing the rates of death and end-stage kidney disease from 50% to less than 15% at 1 to 2 years [[3], [4], [5]]. However, our results suggest that patients with aHUS continue to experience significant impairments in health and function across several domains, which may be driven by a number of aHUS-related factors, comorbidity-related factors, or other patient factors [17].

CKD remains a major comorbidity for aHUS survivors and was present in 60% of our cohort. This rate is consistent with a recent report from the UK National Renal Complement Therapeutics Center, which reported a high prevalence of persistent severe CKD at 5 years from diagnosis, with 46% of patients having CKD stage ≥ 3 and 21% of patients having ESRD [8]. Our study did not find a statistically significant difference in T-scores of PROMIS measures based on the presence of CKD or a need for dialysis at any point, perhaps due to the small sample size. Still, the high prevalence of CKD in our cohort is an important potential confounder. Several studies have employed the PROMIS instruments to assess patient-reported quality of life in patients with CKD and kidney transplant recipients [[18], [19], [20], [21]]. A validation study of the PROMIS-Fatigue Computer Adaptive Test and the FACIT-Fatigue Scale in 198 patients with CKD found that 24% had clinically significant fatigue [19]. In another study, PROMIS instruments were administered to 92 dialysis patients, who demonstrated higher mean ± SD T-scores for fatigue (55.0 ± 9.8) and lower physical function (37.9 ± 7.9), but similar cognition (50.3 ± 10.9), compared with the general population [22,23]. In comparison, our aHUS cohort, the majority of whom had CKD but were not on dialysis, similarly had higher scores for fatigue (59.3 ± 10.7; *P* < .001) and lower physical function (44.6 ± 8.9; *P* < .001), but additionally demonstrated impaired cognitive function (41.4 ± 12.0; *P* < .001).

Participants with aHUS in our cohort also had higher scores for anxiety and depression, and more than half of them had been diagnosed with mood disorders, which could contribute to symptoms such as fatigue, sleep disturbance, and brain fog [[24], [25], [26]]. Indeed, we found that patients with self-reported anxiety or depression had worse scores on all PRO measures tested, suggesting that screening for mood disorders and providing appropriate mental health treatment and support services may mitigate some of these effects [27]. An exploratory qualitative interview study from the Netherlands found that adults with aHUS reported that the emotional toll of aHUS is pervasive and persistent, being characterized by feelings of fear, guilt, and trauma affecting both patients and caregivers [17]. Depression is also a prevalent comorbidity among patients with CKD [23]. The complex interaction between mood disorders, CKD, and aHUS – and their potential contributions to symptoms patients ascribe to aHUS – underscores the need for further research focusing on patient-reported outcomes and optimizing quality of life in chronic diseases.

There may also be additional factors underlying cognitive symptoms in patients with aHUS, such as residual organ injury from acute episodes, and potentially even ongoing organ impairment or organ dysfunction during “remission.” For example, a follow-up study including 93 patients enrolled in any of the 5 registrational trials of eculizumab reported a decline in renal function in 30.1% of patients over a median (IQR) follow-up time of 65.7 (9.9, 102.2) months, and this rate was higher in those who discontinued (40%) vs those who stayed on eculizumab (23%) [27]. In immune thrombotic thrombocytopenic purpura, another type of TMA, recent studies suggest that cerebral ischemic lesions sustained during acute episodes can progress even during hematologic remission, contributing to cognitive impairment [28]. Other studies on immune thrombotic thrombocytopenic purpura suggest that subclinical cerebrovascular, cardiac, and renal disease may be progressive during apparent clinical remission. In the current study, remission status could not be validated using objective laboratory data, such as normalization of platelet counts and lactate dehydrogenase or a percent change in creatinine, and it remains unknown whether patients who reported “good control” were referring to symptomatic improvement or knowledge of objective hematologic or renal parameters. Important variables such as time to remission, relapse dynamics, and treatment strategies are not addressed. These elements will be important in future research to better understand the pathophysiologic basis of patient-reported functional limitations in aHUS.

Our findings are consistent with those of prior studies of PROs in aHUS. Greenbaum et al. [29] reported high baseline levels of fatigue and impairment in the general health status among patients enrolled in the Global aHUS Registry using the FACIT-Fatigue and general health measures at 2 or more time points [29]; a majority of patients who initiated eculizumab after the baseline visit (*n* = 23) achieved clinically meaningful improvements in fatigue, improved general health status, and a 25% to 30% reduction in symptoms of fatigue, weakness, irritability, nausea/vomiting, and swelling over the course of the study [29]. Other studies have also shown improvements in fatigue and health-related quality of life in adults with aHUS treated with eculizumab [30]. Most of these studies have used short measures of global quality of life rather than the PROMIS 29 v2.0 measure, which reliably evaluates multiple domains of quality of life [15]. While our study did not include proxy-reported metrics, interestingly, in a pediatric study, Werner et al. [31] reported that self- and proxy-reported health-related quality of life were not appreciably diminished in school-aged children with aHUS, indicating the need to study different subsets of aHUS patients.

Our study has several limitations. Self-selection or volunteer bias, driven by greater participation of those with more severe disease, may have contributed to an overestimation of the disease burden. Additionally, most respondents in our cohort were White and female, while aHUS does not have a known race or sex predilection, suggesting potential selection bias. The cross-sectional design of our study did not allow for longitudinal evaluation of changes in symptom burden or PROMIS measures over time. However, the median time from diagnosis in our study was greater than 4 years, representing the first assessment of PROs in a cohort with long-standing aHUS. The survey was not structured to capture the clinical context for the discontinuation of complement inhibitor therapy in those who discontinued therapy, which is an ongoing area of interest in aHUS management [[32], [33], [34]]. A qualitative study of international aHUS experts by Germeni et al. [16] was the first to systematically explore the complexity of factors influencing treatment discontinuation decisions in adult patients with aHUS. Most experts favored conditional discontinuation of anticomplement treatment over lifelong treatment, citing treatment burden, high costs, and potential side effects as reasons for discontinuation. Treatment discontinuation was only considered after 3 to 6 months of therapy in patients with steadily improved renal function and a resolved trigger for aHUS (eg, pregnancy), if known. The key points defined by experts in reaching a decision as to whether to discontinue anti-C5 treatment following complete remission included:

• Patient risk stratification into high- or low-risk of relapse according to a number of factors (kidney function, age at first episode, presence of renal transplant, presence of mutations conferring a high risk of recurrence, CKD stage, family history of prior TMA, disease severity at presentation, rapidity of eculizumab response, previous relapse episodes, and extrarenal manifestations).
• Immediate access to retreatment (within 24-48 hours) in the event of relapse, along with available funding for monitoring and retreatment.
• Patient preference and adherence to monitoring.
• Prior negative experiences or concerns about potential consequences of relapse were cited as influencing the decision to continue treatment.

In our cohort, the numbers were insufficient to explore differences in PROs between patients who continued or discontinued complement inhibition therapy. The survey was similarly not designed to evaluate differences in PROs in patients on eculizumab vs ravulizumab vs off-complement blockade. This analysis would be of interest to understand how PROs relate directly to treatment effects. The NIH PROMIS measures have been used and validated for the US population across several disorders, including rare hematological disorders, kidney disorders, and renal transplant recipients [20,21,[35], [36], [37]]. However, it is possible that the PROMIS instruments may not capture all aspects of this rare disease. Patients were invited to write narrative responses about their experience or provide feedback on the survey to mitigate this limitation. The major themes identified from participant feedback were that more emphasis is needed on the impact of CKD and dialysis, caregiver support and mental health resources, and that pain (included in the PROMIS-29v2.0) is not a major concern.

In conclusion, in this first study focusing on PROs in patients with long-standing aHUS, patients reported a chronically impaired global health status with reduced physical and cognitive function and higher levels of fatigue, anxiety, depression, and sleep disturbances compared with the general population. These results should be interpreted with caution, given the prevalence of comorbid anxiety, depression, and advanced renal disease, which may confound symptom attribution to aHUS. Our results, nonetheless, highlight the value of incorporating PRO assessments in clinical trials of novel agents in aHUS and highlight areas of unmet need in clinical practice. Further research is needed to identify modifiable factors contributing to poor functional outcomes, including subclinical disease activity and comorbidities, treatment burdens, and effects.

## References

[1] Afshar-Kharghan V. (2016). Atypical hemolytic uremic syndrome. Hematology Am Soc Hematol Educ Program.

[2] Joseph C., Gattineni J. (2013). Complement disorders and hemolytic uremic syndrome. Curr Opin Pediatr.

[3] Fakhouri F., Schwotzer N., Frémeaux-Bacchi V. (2023). How I diagnose and treat atypical hemolytic uremic syndrome. Blood.

[4] Fremeaux-Bacchi V., Fakhouri F., Garnier A., Bienaime F., Dragon-Durey M.A., Ngo S. (2013). Genetics and outcome of atypical hemolytic uremic syndrome: a nationwide French series comparing children and adults. Clin J Am Soc Nephrol.

[5] Legendre C.M., Licht C., Muus P., Greenbaum L.A., Babu S., Bedrosian C. (2013). Terminal complement inhibitor eculizumab in atypical hemolytic-uremic syndrome. N Engl J Med.

[6] Fakhouri F., Hourmant M., Campistol J.M., Cataland S.R., Espinosa M., Gaber A.O. (2016). Terminal complement inhibitor eculizumab in adult patients with atypical hemolytic uremic syndrome: a single-arm, open-label trial. Am J Kidney Dis.

[7] Dixon B.P., Kavanagh D., Aris A.D.M., Adams B., Kang H.G., Wang E. (2024). Ravulizumab in atypical hemolytic uremic syndrome: an analysis of 2-year efficacy and safety outcomes in 2 phase 3 trials. Kidney Med.

[8] Brocklebank V., Walsh P.R., Smith-Jackson K., Hallam T.M., Marchbank K.J., Wilson V. (2023). Atypical hemolytic uremic syndrome in the era of terminal complement inhibition: an observational cohort study. Blood.

[9] Kavanagh D., Ardissino G., Brocklebank V., Bouwmeester R.N., Bagga A., Ter Heine R. (2024). Forum Participants. Outcomes from the International Society of Nephrology Hemolytic Uremic Syndromes International Forum. Kidney Int.

[10] Rondeau E., Cataland S.R., Al-Dakkak I., Miller B., Webb N.J.A., Landau D. (2019). Eculizumab safety: five-year experience from the Global Atypical Hemolytic Uremic Syndrome Registry. Kidney Int Rep.

[11] Barbour T., Scully M., Ariceta G., Cataland S., Garlo K., Heyne N., 311 Study Group Members (2021). Long-term efficacy and safety of the long-acting complement C5 inhibitor ravulizumab for the treatment of atypical hemolytic uremic syndrome in adults. Kidney Int Rep.

[12] Delaye J., Cacciatore P., Kole A. (2022). Valuing the "burden" and impact of rare diseases: a scoping review. Front Pharmacol.

[13] Bogart K., Hemmesch A., Barnes E., Blissenbach T., Beisang A., Engel P., 311 Study Group Members (2022). Healthcare access, satisfaction, and health-related quality of life among children and adults with rare diseases. Orphanet J Rare Dis.

[14] Basch E., Bennett A.V. (2014). Patient-reported outcomes in clinical trials of rare diseases. J Gen Intern Med.

[15] Hays R.D., Spritzer K.L., Schalet B.D., Cella D. (2018). PROMIS®-29 v2.0 profile physical and mental health summary scores. Qual Life Res.

[16] Germeni E., Cooper J., Briggs A., Laurence J. (2024). Treatment discontinuation in adults with atypical hemolytic uremic syndrome (aHUS): a qualitative study of international experts’ perspectives with associated cost-consequence analysis. BMC Nephrol.

[17] Bouwmeester R.N., Engel L.J., Altena W., Renette C., van Daelen C., van Kempen E. (2024). Living with atypical hemolytic uremic syndrome in the Netherlands: patient and family perspective. Kidney Int Rep.

[18] Tang E., Yantsis A., Ho M., Hussain J., Dano S., Aiyegbusi O.L. (2024). Patient-reported outcome measures for patients with CKD: the case for Patient-Reported Outcomes Measurement Information System (PROMIS) tools. Am J Kidney Dis.

[19] Dano S., Hussain J., Edwards N., Sun Y.I., Li M., Howell D. (2023). Assessing fatigue in patients receiving kidney replacement therapy using PROMIS computer adaptive testing. Am J Kidney Dis.

[20] Selewski D.T., Massengill S.F., Troost J.P., Wickman L., Messer K.L., Herreshoff E. (2014). Gaining the Patient Reported Outcomes Measurement Information System (PROMIS) perspective in chronic kidney disease: a Midwest Pediatric Nephrology Consortium study. Pediatr Nephrol.

[21] Tang E., Ekundayo O., Peipert J.D., Edwards N., Bansal A., Richardson C. (2019). Validation of the Patient-Reported Outcomes Measurement Information System (PROMIS)-57 and -29 item short forms among kidney transplant recipients. Qual Life Res.

[22] Sturgill D.A., Bal N., Nagavally S., Wolfgram D.F. (2020). The relationship between dialysis metrics and patient-reported cognition, fatigue, and physical function. Kidney Dis (Basel).

[23] Bahall M., Legall G., Lalla C. (2023). Depression among patients with chronic kidney disease, associated factors, and predictors: a cross-sectional study. BMC Psychiatry.

[24] Targum S.D., Fava M. (2011). Fatigue as a residual symptom of depression. Innov Clin Neurosci.

[25] Baldwin D.S., Papakostas G.I. (2006). Symptoms of fatigue and sleepiness in major depressive disorder. J Clin Psychiatry.

[26] Alim-Marvasti A., Ciocca M., Kuleindiren N., Lin A., Selim H., Mahmud M. (2024). Subjective brain fog: a four-dimensional characterization in 25,796 participants. Front Hum Neurosci.

[27] Menne J., Delmas Y., Fakhouri F., Licht C., Lommelé Å., Minetti E.E. (2019). Outcomes in patients with atypical hemolytic uremic syndrome treated with eculizumab in a long-term observational study. BMC Nephrol.

[28] Chaturvedi S., Yu J., Brown J., Wei A., Selvakumar S., Gerber G.F. (2023). Silent cerebral infarction during immune TTP remission: prevalence, predictors, and impact on cognition. Blood.

[29] Greenbaum L.A., Licht C., Nikolaou V., Al-Dakkak I., Green J., Haas C.S. (2020). Functional assessment of fatigue and other patient-reported outcomes in patients enrolled in the Global aHUS Registry. Kidney Int Rep.

[30] Mukherjee A.A., Kandhare A.D., Bodhankar S.L. (2018). Evaluation of health-related quality of life in hemolytic uraemic syndrome patients treated with eculizumab: a systematic evaluation on basis of EMPRO. Ren Fail.

[31] Werner H., Buder K., Landolt M.A., Neuhaus T.J., Laube G.F., Spartà G. (2017). Long-term health-related quality of life and psychological adjustment in children after haemolytic-uraemic syndrome. Pediatr Nephrol.

[32] Chaturvedi S., Dhaliwal N., Hussain S., Dane K., Upreti H., Braunstein E.M. (2021). Outcomes of a clinician-directed protocol for discontinuation of complement inhibition therapy in atypical hemolytic uremic syndrome. Blood Adv.

[33] Fakhouri F., Fila M., Hummel A., Ribes D., Sellier-Leclerc A.L., Ville S. (2021). Eculizumab discontinuation in children and adults with atypical hemolytic-uremic syndrome: a prospective multicenter study. Blood.

[34] Acosta-Medina A.A., Moyer A.M., Go R.S., Willrich M.A.V., Fervenza F.C., Leung N. (2023). Complement gene variant effect on relapse of complement-mediated thrombotic microangiopathy after eculizumab cessation. Blood Adv.

[35] Zhang J., Dewitt B., Tang E., Breitner D., Saqib M., Li D. (2021). Evaluation of PROMIS Preference Scoring System (PROPr) in patients undergoing hemodialysis or kidney transplant. Clin J Am Soc Nephrol.

[36] van Hoorn E.S., Willems S.P.E., Al Arashi W., de Moor A.S., van Kwawegen C.B., Teela L. (2024). SYMPHONY consortium and the Dutch research group for PROMIS implementation in inherited bleeding disorders. Psychometrics of patient-reported outcomes measurement information system in von Willebrand disease, inherited platelet function disorders, and rare bleeding disorders. Res Pract Thromb Haemost.

[37] van Balen E.C., Haverman L., Hassan S., Taal E.M., Smit C., Driessens M.H. (2021). Validation of PROMIS Profile-29 in adults with hemophilia in the Netherlands. J Thromb Haemost.

