# Supplementary material for: The Patient-Reported Outcomes Measurement Information System perspective of adults with long-standing atypical hemolytic uremic syndrome
Source: Res Pract Thromb Haemost. 2025 Oct 13;9(8):103224. doi: 10.1016/j.rpth.2025.103224 (PMC12703853; doi:10.1016/j.rpth.2025.103224)
Supplement: Supplementary Tables S1-S4 [file mmc1.docx]

SUPPLEMENTAL DATA

**Supplementary Table S1. T Scores of aHUS patients with and without self-reported aHUS relapse on selected PROMIS instruments**

| **PROMIS measure**  **T score, mean (SD)** | **Self-reported aHUS relapse** | | **P*** |
| --- | --- | --- | --- |
|  | **Yes (N=10)** | **No (N=35)** |  |
| *PROMIS-29v2.0* |  |  |  |
| - Physical function | 39.3 (5.8) | 45.7 (9.0) | 0.036 |
| - Anxiety | 58.3 (10.6) | 59.2 (10.5) | 0.976 |
| - Depression | 54.9 (7.0) | 55.8 (10.1) | 0.973 |
| - Fatigue | 60.6 (5.6) | 58.4 (11.7) | 0.364 |
| - Sleep disturbance | 50.8 (6.4) | 54.1 (7.9) | 0.357 |
| - Pain interference | 52.5 (10.5) | 50.6 (10.3) | 0.729 |
| - Ability to participate in social roles | 42.5 (5.2) | 49.4 (9.5) | 0.099 |
| - Cognitive function abilities | 43.5 (5.3) | 47.9 (7.0) | 0.314 |
| Cognitive function 8a | 43.5 (5.6) | 48.2 (7.1) | 0.562 |
| Social isolation | 51.0 (11.4) | 48.8 (10.3) | 0.673 |

*P values using Mann Whitney U test to compare groups

**Supplementary Table S2. T Scores of aHUS patients with and without self-reported chronic kidney disease on selected PROMIS instruments**

| **PROMIS measure**  **T score, mean (SD)** | **Self-reported diagnosis of chronic kidney disease** | | **P*** |
| --- | --- | --- | --- |
|  | **No (N=20)** | **Yes (N=30)** |  |
| *PROMIS-29v2.0* |  |  |  |
| - Physical function | 44.3 (8.8) | 43.7 (9.1) | 0.857 |
| - Anxiety | 57.4 (8.6) | 60.1 (10.7) | 0.266 |
| - Depression | 54.4 (11.2) | 56.7 (9.1) | 0.765 |
| - Fatigue | 57.7 (11.5) | 60.5 (10.5) | 0.897 |
| - Sleep disturbance | 54.7 (9.4) | 52.9 (6.2) | 0.565 |
| - Pain interference | 50.9 (10.5) | 52.0 (10.1) | 0.990 |
| - Ability to participate in social roles | 48.6 (9.4) | 46.8 (6.5) | 0.644 |
| - Cognitive function abilities | 47.4 (8.5) | 46.7 (6.3) | 0.981 |
| Cognitive function 8a | 41.9 (12.1) | 41.4 (12.3) | 0.931 |
| Social isolation | 41.5 (8.2) | 53.9 (11.9) | 0.556 |

*P values using Mann Whitney U test to compare groups

**Supplementary Table S3. T Scores of aHUS patients with and without self-reported experience of hemodialysis at any point on selected PROMIS instruments**

| **PROMIS measure**  **T score, mean (SD)** | **Ever received hemodialysis** | | **P*** |
| --- | --- | --- | --- |
|  | **Yes (N=36)** | **No (N=14)** |  |
| *PROMIS-29v2.0* |  |  |  |
| - Physical function | 44.9 (9.5) | 43.7 (7.2) | 0.939 |
| - Anxiety | 60.3 (9.8) | 56.3 (9.1) | 0.172 |
| - Depression | 56.1 (9.8) | 55.2 (9.5) | 0.854 |
| - Fatigue | 59.5 (11.6) | 58.7 (8.5) | 0.862 |
| - Sleep disturbance | 53.5 (7.3) | 53.9 (8.3) | 0.957 |
| - Pain interference | 52.1 (10.7) | 50.7 (9.1) | 0.621 |
| - Ability to participate in social roles | 48.3 (10.6) | 46.8 (5.8) | 0.943 |
| - Cognitive function abilities | 47.1 (7.2) | 46.6 (7.2) | 0.970 |
| Cognitive function 8a | 41.6 (13.2) | 40.8 (8.7) | 0.841 |
| Social isolation | 49.5 (11.4) | 50.8 (8.9) | 0.794 |

*P values using Mann Whitney U test to compare groups

**Supplementary Table S4. T Scores of aHUS patients with and without current treatment with hemodialysis on selected PROMIS instruments**

| **PROMIS measure**  **T score, mean (SD)** | **Currently receiving hemodialysis** | | **P*** |
| --- | --- | --- | --- |
|  | **No (N=45)** | **Yes (N=5)** |  |
| *PROMIS-29v2.0* |  |  |  |
| - Physical function | 45.5 (8.7) | 36.2 (4.5) | 0.016 |
| - Anxiety | 58.6 (9.9) | 63.9 (6.9) | 0.203 |
| - Depression | 55.3 (9.8) | 60.4 (6.7) | 0.203 |
| - Fatigue | 58.4 (10.8) | 67.3 (6.2) | 0.055 |
| - Sleep disturbance | 53.3 (7.7) | 56.8 (4.9) | 0.227 |
| - Pain interference | 51.4 (10.3) | 56.1 (9.7) | 0.384 |
| - Ability to participate in social roles | 48.6 (9.6) | 40.7 (4.0) | 0.069 |
| - Cognitive function abilities | 47.1 (7.3) | 46.0 (4.7) | 0.923 |
| Cognitive function 8a | 41.8 (12.1) | 38.1 (12.5) | 0.732 |
| Social isolation | 49.3 (10.7) | 55.6 (8.9) | 0.394 |

*P values using Mann Whitney U test to compare groups
